# Determinants of policy and uptake of national vaccine programs for pregnant women: results of mixed method study from Spain, Italy, and India

**DOI:** 10.1080/21645515.2020.1831858

**Published:** 2020-11-20

**Authors:** Lois Privor-Dumm

**Affiliations:** International Vaccine Access Center, Johns Hopkins Bloomberg School of Public Health, Baltimore, USA

**Keywords:** Maternal vaccination, vaccine, policy, pregnant women, life-course approach, decision-making, implementation, Spain, Italy, India

## Abstract

An important strategy for addressing maternal and newborn risks of disease is through vaccinating pregnant women. We conducted a mixed-methods study including a narrative literature review of drivers of maternal vaccination and key informant interviews in Spain, Italy, and India to characterize different approaches to national maternal immunization programs. Fifty-nine respondents participated in the study conducted between November 2018 and January 2019. Policies in Spain and Italy both reflect a life-course approach to vaccination, but recommendations and how they ensure uptake differs. Italy was focused on tracking of progress and mandates to ensure compliance in all regions, while Spain, an early adopter, relied more on advocacy and building provider acceptance. India includes Td in their national program, but the political will and advocacy for other vaccines are not seen. Needs for improving rates of maternal vaccination include education of health-care providers and pregnant women, use of central registries to track progress, stronger global guidance for use of vaccines, and engagement of champions, particularly obstetrician-gynecologists (ob-gyns). Health security concerns can also be leveraged to build political priority and needed platforms to detect disease and deliver vaccines in some countries. Understanding what drives a country’s maternal immunization program decisions and the success of implementation is useful in designing strategies to share best practices and guide support to strengthen platforms for maternal vaccination.

## Introduction

The global community has committed to ending preventable deaths in newborns and significantly reducing maternal mortality.^1^ An important strategy for addressing maternal and newborn deaths is through immunizing pregnant women to prevent disease in both the mother and the newborn.^2–5^ Pertussis, influenza, and tetanus are three diseases for which vaccines are currently available and recommended for pregnant women. The World Health Organization (WHO) recommends that countries give the highest priority to pregnant women if they are considering expansion of seasonal influenza programs,^6^ but even then, many countries do not have national influenza vaccination policies or recommendations in place, particularly in low- and middle-income settings.^7–9^ In Europe, where a goal of 75% influenza vaccination coverage of key risk groups including pregnant women has been established, no country achieved this target in the 2017/18 season and many countries did not report coverage.^10^ In 2017, WHO updated tetanus toxoid (TT) recommendations to include tetanus-diphtheria (Td) vaccines for pregnant women,^11^ and has a permissive recommendation for acellular pertussis-containing vaccines in pregnant women (e.g, national programs may consider 1 dose of tetanus, diphtheria, acellular pertusis (Tdap) in the 2^nd^ or 3^rd^ trimester in addition to vaccination of infants in high or increasing infant pertussis morbidity or mortality).^12^ Nonetheless, a number of countries, including Italy,^12^ Spain,^13^ nine other countries in Europe,^14^ United States (US),^15^ New Zealand, Australia, Argentina, Brazil, the Bahamas, Chile, Costa Rica, El Salvador, Honduras, Mexico^16^ have recommended Tdap vaccines in pregnancy, albeit at different schedules. Many countries report low coverage for Tdap in pregnant women^17–20^ and some have no reliable coverage data.^21,22^

There are a number of reported reasons for the low coverage rates for maternal vaccination. Safety is reported to be a leading concern amongst both pregnant women and providers in a number of countries.^23,24^ Provider recommendation was a predictor of acceptance in a number of countries.^23,25^ It is influenced by a series of risk-benefit determinations including financial and practical considerations, perception about the value of vaccine, and risks and steps taken to promote the benefits of vaccination.^26,27^ There is still a need to improve awareness of vaccine benefits and risks amongst providers^28^ and pregnant women.^21,27,29^ Lack of perceived need for maternal vaccination and low awareness about the dangers of VPDs in pregnancy also leads to low rates of acceptance.^30^

Despite an understanding of the barriers seen across countries, there is still little understanding of the potential similarities and differences between countries. Better understanding the varying approaches toward maternal vaccination across countries can help in developing strategies to address similar issues and share best practices across countries. To determine if there were unique characteristics of countries’ maternal vaccination programs, we examined three countries in more detail.

## Methodology

The project used a mixed-methods approach including a narrative literature review, desk research, and qualitative interviews to develop case studies to examine key factors influencing decision-making and uptake of maternal immunization.

### Country selection

We selected three countries – Spain, Italy, and India – to provide a diversity of situation, approach, and performance in order to compare and contrast. Spain and Italy were selected to better understand countries with similar recommendations for vaccinating pregnant women but for different drivers of decisions and differing levels of uptake. India was selected to determine how a country's more limited national recommendations for maternal vaccination approached decision-making and implementation differently.

### Narrative literature review and desk research

We conducted a narrative literature review and desk research to identify barriers and drivers of decision-making and implementation for national government programs to inform a semi-structured interview guide used in the qualitative component of the study. Previous research on drivers of vaccine introduction and uptake^31,32^ also informed our research. We used the following search terms: “vaccination”[tiab] OR “immunization”[tiab] AND (“maternal immunization”[tiab] OR “maternal immunisation”[tiab] OR pregnan*[tiab]) AND (“implement*” OR “introduc*” OR “recommend*” OR polic* OR barrier OR gap OR challenge) AND (Spain OR Italy OR India). Results were limited to those published between 2005 and 2019, resulting in 237 articles. To further identify relevant articles, the references lists of pertinent articles were examined and any additional relevant articles were included. Inclusion criteria included 1) full-text availability; 2) published within the last 15 years; and 3) articles with a focus on maternal vaccination policies, barriers, and implementation. Exclusion criteria included articles that merely focused on morbidity and mortality associated with maternal immunization, without a focus on policy, barriers, or implementation. We scanned gray literature including government, partner, and professional association websites, media and other reports for country background, vaccine recommendations and other public policies, reports on vaccine uptake, and maternal vaccine stakeholders. Searches were conducted in English, Spanish, and Italian and supplemented by Google Translate.

### Interviews

We used a snowball approach to identify key informants in each country comprised of experts from a vaccine technical, maternal health, program implementation, health economics & financing, and advocacy backgrounds. One-hour interviews were conducted in-person or by phone in English. Interviews were recorded and transcribed. No identifiers were included in the transcriptions to maintain confidentiality, only expertise and job type categorization. The Johns Hopkins Bloomberg School of Public Health Institutional Review Board deemed this to be non-human subjects research.

### Case study analysis

For the qualitative component of the study, a thematic analysis was performed on the interviews using ATLAS.ti to create a case study for each country. This data was plotted, and graphs designed using Graph Pad Prism Version 7.0. Tables were also developed, and results were compared across countries.

## Results

**Figure 1.**
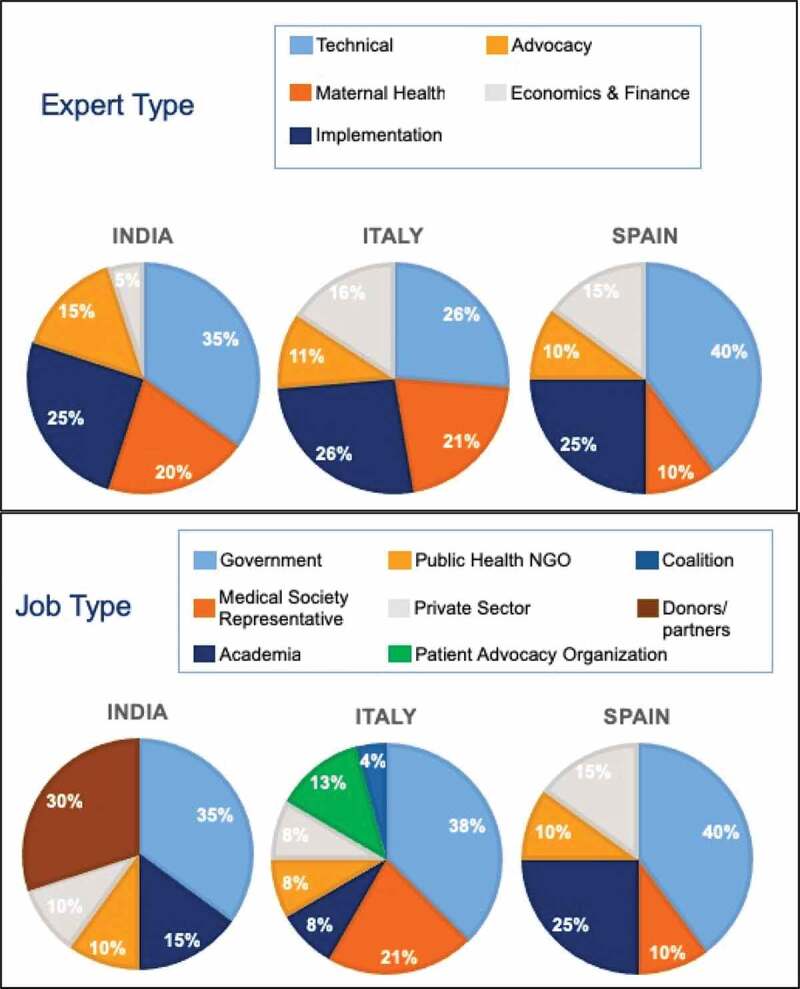
Profile of key respondents interviewed. n = 20 in Spain and India, n = 19 in Italy. Some respondents categorized into multiple job types in Italy

We reviewed 237 articles identified in the literature review, which covered the following themes: decision-making process; priority of life-course approach to vaccination, maternal vaccination infrastructure including processes, registries, and implementation; key players and stakeholders in decision-making and implementation; access to maternal immunization and degree of vaccine hesitancy; financing; and system-level barriers and facilitators of maternal vaccination. We conducted 59 key informant interviews (20 per country in Spain and India and 19 in Italy) November 2018 and January 2019, probing on the themes listed above. Results of the literature review, desk research, and interviews are shown below. The distribution of respondents is listed in Figure 1.

### Decision-making and recommendations

#### Recommendations

Both Spain and Italy had recommended influenza vaccines and Tdap in pregnancy.^14^ Spain recommends Tdap from 27 weeks and influenza vaccine in any trimester,^33^ since 2014^19^ and influenza vaccination since 2005.^34^ In 2017, Italy recommended Tdap at 27–36 weeks and influenza vaccine in the 2^nd^ or 3^rd^ trimester.^35,36^ In 2019, Italy updated influenza vaccine recommendations to any trimester.^37^ India, a large lower-middle-income country, only recommended TT and had recently updated their recommendation to Td as part of the national program.^38^ India does not nationally recommend influenza vaccine for pregnant women, even for pandemic preparedness.^39–41^

#### Drivers of decisions

Based on findings both from the literature review and respondents, the main drivers of decision-making varied in each country (Table 1). For example, in India, low cost of TT/Td vaccine was cited as an enabler decisions and political priority (driven by a high infant mortality rate). Respondents in India also noted that other maternal vaccines had not been considered, likely because of cost, lack of advocacy and poor data. Italian respondents cited newborn mortality as the most frequent driver of national decisions to adopt vaccine while Spanish respondents frequently noted recommendations from other countries and wanting to be known as a leader in the region. Both Italy and Spain had a recent focus on the life-course as reflected in their national calendars. Another frequently mentioned factor in Spain was the shortage of adolescent diphtheria-tetanus-acellular pertussis vaccine and availability of Tdap.^42,43^ Lack of safety data in pregnant women was not mentioned as a driver of decision-making by respondents in any country. When prompted further, respondents noted its importance, but did not view this as preventing decisions. Several Indian respondents cited vaccine hesitancy as a barrier. A few respondents in India mentioned an overall lack of disease burden data in decision-making for pregnant women. In Italy, elections and the influence of a female physician's health minister were also mentioned as a driver of decisions.

**Table 1.** Frequently cited drivers of maternal immunization decision-making

India	Italy	Spain
Cost	Newborn protection	Other Country Decisions
Infant mortality rate	Life course vaccination	New adult vaccine calendar implemented 2017/2018
Prime Minister priority	Harmonization of regional vaccine calendars	Shortages in pediatric diphtheria, tetanus, acellular pertussis (DTaP) but not maternal Tdap
Maternal mortality rate	Disease burden	Disease burden
Neonatal disease burden	Mandatory pediatric vaccinations	Safety
		Cocooning
		Cost-effectiveness

#### Stakeholders involved in decision making

**Figure 2.**
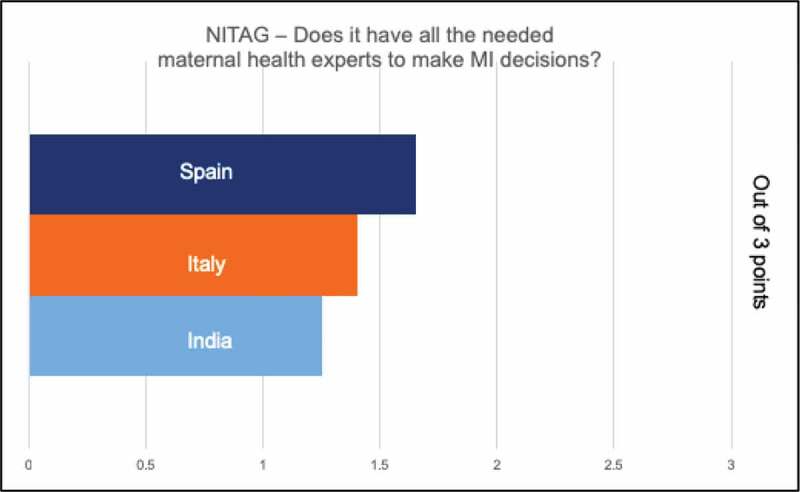
Respondent ratings of presence of maternal immunization expertise on NITAGS. 0 = no presence of experts on NITAG/expert working group; 1 = one representative; 2 = 2 representatives; 3 = 3 or more experts on NITAG/expert working group

All countries had centralized decision-making in the form of a technical advisory group (NITAG), which respondents noted was often comprised of pediatricians. In India, some respondents relayed a concern that the voice of the ob-gyns was not strong despite their presence on the National Technical Advisory Group on Immunization (NTAGI). Most respondents in all countries did not feel there was a champion for maternal immunization to move the agenda forward. In general, respondents perceived the level of specific expertise in decision-making for pregnant women to be limited. When asked to rate adequacy of maternal immunization expertise for decision-making, Spanish representatives rated this highest, Italy next, and India lowest, noting only 1–2 representatives on the NITAG (Figure 2). In addition, in India, lack of data in pregnant women was a frequently cited barrier to decision-making, including for “maternal immunization experts.”

#### Health security

Health security and outbreaks were mentioned by respondents in all countries, although not necessarily in response to a question regarding facilitators for decision-making. In India, the H1N1 outbreak did not result in a recommendation to adopt pandemic influenza vaccines (although distributed to health workers) and one respondent told us that pregnant women were not considered in the conversation. According to respondents in Spain, pertussis outbreaks drove a decision to act according to respondents and in Italy, outbreaks were reported by some as a driver of new or expanded policies.

### Implementation: facilitators and barriers

#### Advocacy & provider buy-in

Advocacy was described as a prime facilitator of uptake in Spain and India. While health providers are viewed as important influencers of decisions and uptake, Spanish physicians were not consistently convinced on the need for vaccine. The lack of physician buy-in amongst some was noted as a significant challenge in Spain. In India, maternal health advocates and those representing marginalized populations (e.g., WHO, UNICEF, non-governmental organizations) were important, but many commented that providers, in general, were not making the recommendation, perhaps because they are not convinced due to a lack of local studies of vaccines in pregnant women. Some respondents questioned whether maternal health was a priority in India. Generally, ob-gyns were cited has having a strong influence on implementation at an individual level, but were not correspondingly viewed as strong champions at a national level.

#### Registries and monitoring in a centralized manner

**Figure 3.**
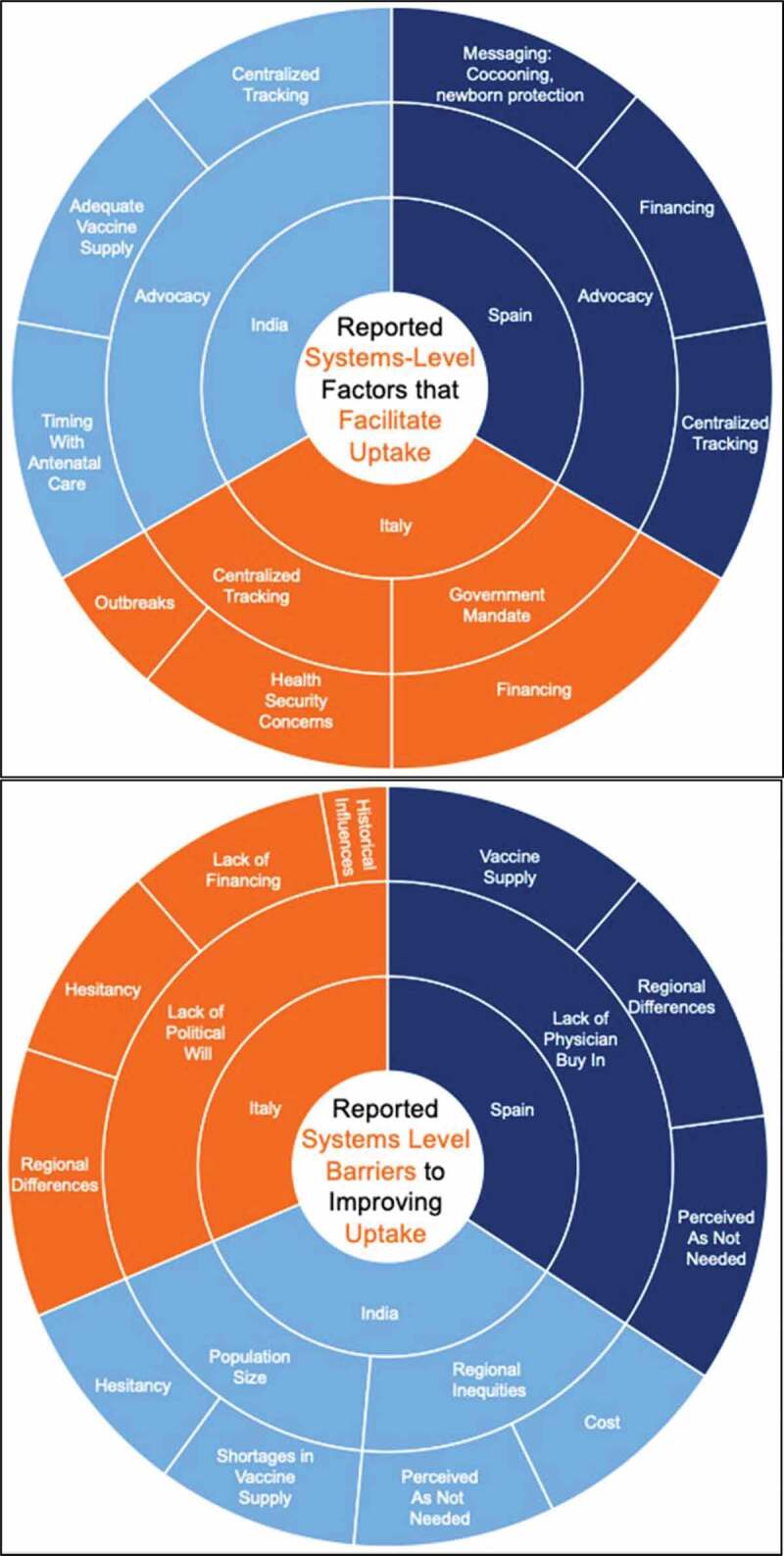
System-level facilitators and barriers to improving uptake. Key: Inner circle: Factor mentioned by ~100% of respondents, Outer Circle: Other mentioned factors, Size is proportional to frequency that respondents mentioned it as a factor. Advocacy is referring to pediatric and new-born health experts advocating for vaccines to protect baby and mother. Ob-gyn’s seem to not play an influential role in uptake currently in the case study countries

In Italy, respondents credited registries and mandates for their program’s ability to achieve high uptake (Figure 3). Centralized tracking of coverage rates was mentioned by some respondents in all three countries as a facilitator for uptake. In Italy, respondents spoke about a big push to centralize previously regional vaccination calendars, implement national registries and mandate vaccination to ensure consistency across regions. Vaccines are mandatory for children and fines are levied for noncompliance. Although the same does not apply to adults, there is a requirement for the regions to offer vaccines. By contrast, a smaller number of respondents in Spain also suggested some degree of centralization through tracking of all regions. Vaccinations in Spain are not mandatory. A recent law in Spain (2016) focuses on disparities across regions and should help reduce some of the differences in coverage rate. Respondents in India explained that there were pilots to create electronic health records (EHRs), which could become part of a centralized dashboard, but they have not currently progressed. Some respondents also commented that the program for maternal immunization is implemented by the National Health Mission, yet decisions and procurement decisions were made under the Universal Immunization Programme (UIP). A couple of respondents mentioned that better coordination was needed.

#### Regional differences and inequities

Regional differences in implementation were mentioned in all three countries, although most common in India, where the concept of inequities was brought up frequently. This was not necessarily referring to TT, which enjoyed a very high coverage rate. Respondents in India spoke of the challenges in antenatal care (ANC), which was the responsibility of the National Health Mission and immunization, a part of the UIP. A few respondents recognized a need for greater coordination between the two programs, noting greater coordination would be essential when vaccines required specific timing. A few respondents noted that there were initiatives to help address inequities such as Intensified Mission Indradhanush (IMI), which has helped improve coverage, monitoring, and surveillance capacity in underperforming districts.^44^ This initiative and focus on pregnant women helped India achieve 81% coverage of TT vaccine in 2018.^45^ In Spain, although respondents were not aware of reliable coverage estimates, administrative data show high national average coverage for Tdap (79–80%in 2017 and 2018), and regional variation ranging from 57% to 93% in 2018.^46^ Studies on acceptance and prescribing, however, suggest coverage rates of Tdap or influenza would be lower.^47–49^ Italy reports on influenza vaccine coverage for pregnant women, but rates are extremely low.^10^ Additional surveys report similarly low coverage and suggest that education and a lack of provider recommendation are important factors in low uptake.^50,51^

#### Financing and cost

Perception of affordability at the patient- and systems-level can be a major barrier to implementation and uptake. All countries reported cost as being influential, to some degree, in successful decision-making and implementation performances. In all three countries, if the vaccine is recommended, it is free for the patient and there are no out-of-pocket costs if the vaccine is received at a designated vaccination place.

India, more frequently than the other two countries, reported the cost of maternal vaccines as a main barrier to adopting and implementing new vaccines. Indeed, respondents reported that tetanus was recommended because of its low cost. Some respondents expressed that pertussis vaccination has not been brought to the agenda due to cost. They also perceived this to be the issue with influenza vaccination. Other respondents noted there was insufficient disease burden data to support decisions, however. Respondents reported that both access and affordability drive differences in uptake that are geographical, regional, rural-urban, poor-rich, and gender-related.

Respondents in Italy described the financing as both a facilitator and barrier to maternal immunization, noting an increase in spending for a new life-course vaccination plan, and cost-effectiveness data that showed that this spending would bring significant savings. However, cuts to healthcare needs and provider staffing resulting from austerity measures were reported by respondents as a potential concern regionally. Regional authorities expressed concerns that some areas may face rising demand for services under the new policy, while simultaneously struggling with declining numbers of physicians.

In Spain, respondents noted strong support for public financing of vaccination. Public financing of vaccines in calendar started in 2006 when the current menu of services offered to all under the Sistema Nacional de Salud.

#### Supply

Some respondents noted that vaccine supply played an important role in maternal immunization. In Spain, maternal pertussis immunization increased following shortages of pediatric DTaP vaccines (the country experienced pertussis outbreaks).^30^ This contributed to the recommendation and political support for maternal Tdap.^32^ In India, several respondents noted the size of the population played a role due to concerns over whether the country would even be able to supply vaccine.

#### Vaccine communication and hesitancy

Hesitancy was noted by some respondents as a barrier to uptake for both women and providers in India^30,52,53^ and Italy.^54–56^ Hesitancy, although seen in Spain,^47,48^ did not emerge as a major barrier. The respondents mentioned the country also had a strong promotion of vaccination which could explain lower perceived levels of vaccine hesitancy. Some Spanish respondents also stated that lack of uptake could be ascribed to a lack of awareness. The issues with hesitancy described in Italy may stem from institutional distrust and political parties which tend to stoke these sentiments. Respondents, however, claimed that distrust and hesitancy around immunization died down during outbreaks. Regional variation in hesitancy was also reported and, in every country, respondents noted the important role of providers, particularly ob-gyns in convincing women to be vaccinated, although most felt they needed to be more proactive and that their influence could be strengthened.

#### Providers and access

Having easy access to vaccination providers can influence level of uptake. Physicians in Spain reported a lack of support at the administrative/implementation level and one respondent noted that Spain spends less on public health and prevention than other countries in the EU. Women do not always know where to access the vaccines as well. In Italy, the view that physicians have a lot of power was common, and due to regional variations, the value placed on vaccines may result in lower coverage rates in some places. The ob-gyns and general practitioners were named as important sources of information and pregnant women could get vaccine free of charge in any national clinic and in pharmacies for the case of influenza vaccine. In India, respondents remarked that women receive vaccines at antenatal care visits and through supplemental immunization activities (SIAs), particularly in high-risk districts. This, combined with a number of other strategies, helped India achieve maternal and neonatal tetanus elimination. There was near-universal mention of this as a source of pride, but some respondents felt that giving other maternal vaccines may still be a challenge if they must be given at a particular time in pregnancy.

## Discussion

Both Italy and Spain appear to be vying for leadership in Europe in terms of their decision-making around life-course immunization, which has become an important driver in decisions around maternal immunization. Italy’s decision-making may have been facilitated by a strong champion, while implementation seems to rely on centralization of the vaccine program, legislation,^57^ and tracking to ensure that years of disparity and hesitancy enabled by differing politics are eliminated. Tdap and influenza vaccines have only been offered to pregnant women since 2017.^36,57^ Awarenessamongst many providers and pregnant women is generally low, indicating significant opportunity for improvement.^58^ Further, safety, while not shown to be a significant barrier for inclusion of existing vaccines in national programs, is a key driver of uptake amongst pregnant women, and will be an important driver for acceptance.^54,58^

Spain, on the other hand, has been an early adopter of immunization, reportedly following the United Kingdom, US, and Australia, as well as looking to Italy. Advocacy appears to be an important driver of vaccine decisions and uptake appears to strong, in at least some places, driven by provider recommendations and a high degree of parental trust in providers. Health security concerns (e.g., pertussis outbreaks) have also helped drive action and reinforced the need to further strengthen their system.

India, by contrast, has not adopted either influenza or pertussis vaccines for pregnant women,^59^ instead following WHO recommendations for tetanus immunization (Td vaccine). Indian respondents were proud of achieving maternal and neonatal tetanus elimination and high rates of immunization coverage in pregnant women.^60^ Despite high neonatal priority and political priority for maternal health, maternal vaccines other than Td, however, are not on the political agenda.^61^ Even during the H1N1 outbreak in 2009, NTAGI did not consider vaccination of pregnant women and deployed vaccine only to health workers.^62^ Respondents observed few Indian champions for maternal immunization, although there is a representation of the Federation of Obstetrics and Gynecologic Societies of India (FOGSI) and maternal health experts on NTAGI. Stronger advocacy efforts, including at the state level, are likely needed to move the agenda, which can include not only consideration of new vaccines, but the data that will be necessary to make decisions about new vaccines in pregnant women. Although safety was not mentioned by respondents in this study as a barrier to vaccine recommendations in India, previous experience indicates disease burden, vaccine safety and efficacy, and economic evaluations will all be needed to move toward a decision.^63,64^

For countries such as India, where decisions have not been made to adopt maternal vaccines beyond Td, sharing experiences about the benefit of maternal immunization programs in other countries, may be useful. Stronger global policies around influenza and pertussis vaccination will likely be needed for countries to adopt them, but the first steps of improving surveillance and developing infrastructure that enables stronger integration with antenatal care will be needed at early stages.^22^ Additionally, global tracking of progress in both recommendations and implementation can help create global.prioritization. Local data will also assist in making the case to policy-makers; however, it should be of high quality and not delay action if a reasonable case can be made using regional data.

Ob-gyns and midwives are important sources for advice, but their voice at the national level for maternal immunization may not be fully leveraged. Potentially, this could be addressed through a deliberate effort to engage ob-gyn and midwifery leadership in the NITAG discussions and empower them to speak up through planned interactions with government and scientists to advocate for needed local data and evidence informed decisions. An organized voice and global and local champions can help raise political priority and action across all countries.^65^

### Limitations and strengths of this study

This study was conducted with a small number of countries and the findings may not represent the full extent of drivers and barriers for maternal immunization. Although we were able to compare and contrast decision-making and implementation in study countries, this does not enable us to describe potential country archetypes. The study does provide, however, important insights upon which further research can be based to understand potential strategies for addressing gaps or leveraging certain approaches to improve vaccine decision-making and uptake.

## Conclusion

Understanding what drives a country’s maternal vaccine program decisions, whether implementation is successful or faces challenges, is useful in designing strategies to share best practices and guide support to strengthen platforms for maternal vaccination. Champions are important to build confidence in maternal vaccination programs and understanding of the need for platforms to educate both providers and expectant mothers, deliver vaccines, and accurately track vaccine uptake. Health security concerns, goals of reducing newborn mortality, or broader life-course immunization and health goals can be leveraged where appropriate. Global monitoring of progress and stronger global guidance to strengthen platforms for maternal vaccination according to the needs of the country may help raise political priority.
